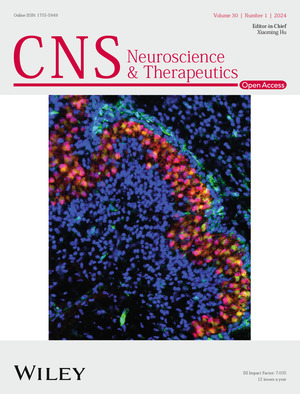# Additional Cover

**DOI:** 10.1111/cns.14622

**Published:** 2024-01-28

## Abstract

The cover image is based on the Original Article *IFN‐γ promotes radioresistant Nestin‐expressing progenitor regeneration in the developing cerebellum by augmenting Shh ligand production* by Jian Hu et al., https://doi.org/10.1111/cns.14485.